# Third-Line Antiretroviral Therapy: What Do We Do When the Appropriate Formulations Are Not Available?

**DOI:** 10.3390/children9040473

**Published:** 2022-03-30

**Authors:** Lisa Jane Frigati, Helena Rabie

**Affiliations:** Department of Paediatrics, Child Health Stellenbosch University, Tygerberg Hospital, Cape Town 7505, South Africa; hrabie@sun.ac.za

**Keywords:** darunavir, dolutegravir, crushing, antiretrovirals

## Abstract

Children on antiretroviral therapy have limited options, particularly if they are failing therapy and live in resource-poor settings. We describe three cases where children accessed third-line antiretroviral therapy off-label, or used them extemporaneously with successful outcomes. We then review the evidence for performing this measure. There is an urgent need for appropriate formulations to treat young children who require a third-line or salvage regimen.

## 1. Introduction

Children that start antiretroviral therapy (ART) at a very young age are often infected with resistant HIV virus. In addition, they may be severely ill, may not have access to optimal regimens and fixed drug combinations, and they often do not tolerate drugs, especially lopinavir/ritonavir (a protease inhibitor (PI) commonly used in standard first-line ART). In addition, common comorbidities such as chronic diarrhea and gastro-esophageal reflux decrease their ability to ingest and absorb medication. Drug–drug interactions can also result in inadequate ART drug levels. These factors, in addition to poor adherence, may result in resistance and ART failure at a young age. Globally, it is estimated that only 64% of children and adolescents are suppressed after 12 months on ART [1].

A review of children failing first-line ART in resource-poor countries showed that 54% had major PI mutations [2]. Children have also been shown to develop resistance to lopinavir/ritonavir faster than adults [3]. There is limited data on how best to treat these children, and limited third-line ART formulations are available. Many of the drugs are not licensed, expensive, or are only available for compassionate use or in solid formulation.

At the time when the children in this case report started ART in the Western Cape province of South Africa, the “Western Cape Consolidated Guidelines for HIV Treatment: Prevention of Mother-to-Child Transmission of HIV (PMTCT), Children, Adolescents and Adults” defined first-line ART for children older than 1 month and weighing more than 3 kg but less than 20 kg, as Abacavir (ABC) + Lamivudine (3TC) + Lopinavir/ritonavir (LPV/r). Children older than 3 years and more than 10 kg were started on ABC, 3TC and Efavirenz. Second-line regimen was only used when children had failed an Efavirenz-containing regimen, and consisted of Abacavir/Zidovudine + Lamivudine + Lopinavir/ritonavir. If a child had been on a PI-based regimen for at least 2 years with virological non-suppression defined as at least three viral load measurements of ≥1000 copies/mL (≥log 3) or VL > 1000, with evidence of clinical or immunological failure, a genotypic resistance test was performed.

If virological non-suppression was present but the child had been on PI- based ART < 2 years, a genotypic resistance test was performed (using an in-house method for integrase inhibitors and a commercial kit for protease inhibitors) if there was a history of intolerance or poor adherence, non-boosting of a PI-based regimen, or no dose adjustment of protease inhibitors to overcome a drug interaction. The results of the resistance test were then referred to the third-line ART committee. The committee is made up of pediatric infectious disease specialists and virologists that review the previous clinical and ART regimen history, as well as the laboratory results along with the adherence and family circumstances. Recommendations are then made for the most appropriate regimens.

Third-line usually refers to a salvage regimen that contains an integrase inhibitor and a next generation protease inhibitor such as Darunavir (DRV) [4,5,6].

The aim of this paper was to describe three cases of children who started on extemporaneous or off-label drugs commonly used in third-line regimens, and describe the evidence for this.

## 2. Materials and Methods

We performed a folder review for each patient, as well as a review of the literature and product inserts.

## 3. Results

### 3.1. Case 1

RN is a girl who started on abacavir, lamivudine, and lopinavir/ritonavir as a standard, first-line, PI-containing regimen at 4 months of age. She had no prior ART for the prevention of mother-to-child transmission, as the mother was unaware of her diagnosis. She had difficulty tolerating the lopinavir/ritonavir and genotypic resistance testing performed at 3 years and 8 months of age showed significant resistance to lopinavir/ritonavir. At the time she weighed 30 kg (obese), but could not swallow whole tablets. She was started on zidovudine, lamivudine, raltegravir, and darunavir with ritonavir. Initially, she was commenced on the darunavir oral suspension (100 mg/mL). This was only available in South Africa on compassionate-use access from the manufacturer by named patient approval from the local regulator. This limited her care to a tertiary institution where clinicians and pharmacists had the resources to ensure drug supply could be maintained. As the parents could not afford to continue travelling to the tertiary institution, it was decided that her parents would crush the darunavir. RN has remained virologically suppressed since 2016, and her weight has remained stable. She was able to transition to combination tablets with tenofovir, lamivudine, and dolutegravir as a fixed-dose combination as a therapy simplification.

### 3.2. Case 2

PGL, now an 8-year-old boy, started abacavir, lamivudine, and lopinavir/ritonavir at 3 months of age. He has spastic diplegia due to HIV encephalopathy. His mother was not virologically suppressed, and had extremely difficult social circumstances. At 4 months of age, he was admitted to hospital with severe acute malnutrition and diagnosed with tuberculosis (TB). He was started on rifampicin-based anti-TB treatment and ritonavir was added to lopinavir/ritonavir to overcome the interaction between lopinavir and rifampicin, according to national guideline recommendations. During this admission he had chronic diarrhea. He was placed in a children’s home due to his social circumstances. At two years of age, he still had not achieved virological suppression and resistance testing showed lopinavir/ritonavir resistance. At this point, his weight was 10.9 kg and he was just under two years.

He was started on zidovudine, lamivudine, darunavir, and ritonavir, despite the fact that he was less than 3 years of age. Initially, he was commenced on the darunavir oral suspension (100 mg/mL), but due to the same reasons as described in the first case we switched to crushing the darunavir. PGL remains virologically suppressed and clinically well, with no further deterioration of his neurological status.

### 3.3. Case 3

CB started on abacavir, lamivudine, and lopinavir/ritonavir from 2 months of age. His mother reported difficulties administering ART and his viral load remained persistently high for the first 2 years of life. Both his parents were virologically supressed at the time he was referred to our clinic. He was also diagnosed with HIV encephalopathy, nephropathy, and gastro-oesophageal reflux. Resistance testing performed at around ten months of age revealed high-level resistance to lamivudine, nevirapine, and efavirenz, but showed no protease inhibitor resistance. Repeat resistance testing performed a year later showed high-level resistance to all protease inhibitors except darunavir and tipranavir (Table 1). His therapy was changed to lamivudine monotherapy in order to prevent the development of further mutations, as there was limited availability of drugs. After he deteriorated clinically, zidovudine, lamivudine, and raltegravir were initiated. He was admitted to a children’s home for 3 months, during which time he was suppressed virologically, but after returning back to his family his viral load was high again, 3 months later. Repeat resistance testing then showed high-level resistance to raltegravir. Thus, at the age of three years he had progressive HIV encephalopathy and four drug class resistance. After broad consultation, we initiated a crushed solid formulation of darunavir with ritonavir, zidovudine and lamivudine, twice-a-day, and we used adult 50 mg film-coated dolutegravir twice daily (there was insufficient data on dolutegravir use in children at that time and no pediatric formulations were available). He has been continuously suppressed since his change in regimen, and his CD4 percentage has increased (Table 2).

## 4. Discussion

These three case studies highlight the complexity of young children that need third-line or salvage regimens. In each case, the drug was prescribed either off-label, as in the case of Case 2, (drug was not licensed for use at less than 3 years of age), or it was given extemporaneously, with the parents having to crush it and dissolve it in water and administer it orally. The clinical and virological outcomes were good, but the case series is limited by lack of therapeutic drug monitoring.

There is a history of ART being given extemporaneously or off-label for children. An example of this is stavudine. Stavudine was part of first-line therapy for adults and children. In South Africa, only the adult formulation was available. Many clinicians that treated children who were unable to swallow capsules or who required a lower dose opened stavudine capsules and dispersed the contents of the capsule in water and then gave the appropriate weight-based dose [7]. Subsequently, pharmacological and pharmacokinetic evaluations using high-performance liquid chromatography confirmed the accuracy of this dosing method for stavudine, and showed that plasma drug exposure after stavudine administration as a “solution” in water was bioequivalent to intact capsule administration [8].

Darunavir (DRV) has been shown to be effective in protease inhibitor-experienced children and has a low side-effect profile. In the ARIEL trial, 56% of children on darunavir achieved virological suppression at 24 weeks, and 81% by 96 weeks [9]. Liquid darunavir was used in children that could not swallow in the study. There are no data on darunavir dosing in children younger than 3 years, because toxicity and mortality were observed in juvenile rats. In a juvenile toxicity study where rats were directly dosed with darunavir (up to 1000 mg/kg), deaths occurred from post-natal day 5 at plasma exposure levels ranging from 0.1 to 1.0 of the human exposure levels. In a 4-week rat toxicology study, when dosing was initiated on post-natal day 23 (the human equivalent of 2 to 3 years of age), no deaths were observed with a plasma exposure (in combination with ritonavir (RTV)) two times the human plasma exposure levels [10]. Oral suspension is recommended for children between 10 and 15 kg. Oral suspension is available in South Africa only through Section 21 authorization (this is a process that enables practitioners to access unregistered medicine within South Africa, the drugs are then imported for the particular child).

Experience of use of protease inhibitors in children is mostly with lopinavir/ritonavir.

The administration of crushed adult 200/50 mg lopinavir/ritonavir tablets to children significantly reduced lopinavir and ritonavir exposure with a decrease in AUC by 45% and 47%, respectively [11]. Therefore, the use of crushed lopinavir/ritonavir tablets should be avoided [11]. However, since darunavir tablets are not formulated as an extended-release formulation, no potential problem is anticipated if the tablets are chewed or crushed.

The evidence for crushing and administering darunavir orally or via an orogastric, or nasoduodenal tube comes from four case reports in adults [12,13,14]. The first patient who had candida esophagitis and dysphagia took crushed darunavir/ritonavir (600/100 mg) achieved through levels of 6.95 ng/mL, measured at 1-month post-treatment initiation, and achieved viral suppression. The second patient had a permanent gastric tube and had two plasma levels measured that were in the therapeutic range with subsequent viral suppression [12]. Another case report describes a 43-year-old man who received the same dose via a nasoduodenal tube and had a serum darunavir concentration of 6.1 at 2 h post-dose and 5.7 at 6 h post-dose [14]. Using these case reports, we concluded that crushing darunavir would be acceptable. In addition, for children more than 15 kg, the dose of liquid and solid formulations of darunavir is the same, suggesting that the PK is not formulation-dependent. We therefore concluded that we could approximate the dose for children 10–15 kg using tablets. It is not clear if 3 years would be the appropriate cut-off to avoid toxicity, as there are no clinical data in younger children; the recommended dose is approximately 20 mg/kg.

Though crushing the tablets solves the issue of access to liquid formulations, children will still need access to ritonavir or cobicistat so that darunavir can be used, and many countries have limited access to solid formulations of ritonavir that are appropriate for children.

Dolutegravir (DTG) 50 mg FC tablets are the only single formulation of DTG currently available in South Africa. The 10 mg dispersible tablets have been licensed by the Food and Drug Administration (FDA), but are still awaiting approval by the South African Health Products Regulatory Authority (SAPHRA) [15]. The South African ART guidelines recommend DTG only for children weighing more than 20 kg. In the past, raltegravir was used in children as part of third-line regimens. For Case 3, with the data that was available for DTG dispersible tablets (DT) 25 mg OD from IMPAACT and ODYSSEY, in this age group, the troughs were broadly in range or even low compared to adult data [16]. The bioavailability equivalence of the dispersible tablet (DT) to the film-coated (FC) tablet is 1:1.6 (therefore, 30 mg DT is approximately 50 mg FC), so it was postulated that 50 mg BD would not lead to extremely high plasma concentrations.

## 5. Conclusions

Clinicians treating children with HIV have a long history of adapting adult and solid formulations to ensure adequate access for children. These case reports illustrate the complexities of adapting limited formulations, but also highlight the urgent need for appropriate formulations of ART for young children. Children who have failed ART need simpler regimens that are easy to administer, and they require us to provide this care close to their homes. Though these examples illustrate the possible success of adapted therapies, these children deserve appropriate formulations—for example, dispersible tablets—as this is often their last potential regimen.

## Figures and Tables

**Table 1:** Prior regimens, mutations, and pre- and post-third-line CD4 and viral load.

	Patient RN	Patient PGL	Patient CB
Regimens prior to resistance test	ABC + 3TC + LPV/r	ABC + 3TC + LPV/r	1. ABC + 3TC + LPV/r 2. AZT + 3TC + RAL
NRTI mutations	L74V, M184V, V35T, E36A, T39A, E40D, S48T, A98S, K122E, S162C, K173T, Q174K, D177E, I178L, T200A, Q207E, R211K, V24K, E240D, D250E, I93L	K70E, M184V, D67D/G	L74V, L74+, M184V, Y115E
NNRTI mutations	none	K103N	V106M, V179D
PI mutations	M46I, I84V, L10M, G17D, M36I, R41K, K55KR, R57K, Q61H, L63A, C67E, H69K	I54V, M46I, V82A, L10LF, K20T	M46I, V82A, I54MV, L10F, K20T
II mutations	ND	ND	T97A, Y143R
CD4 * pre-third-line	1034	38.0	21.5
CD4 * post-third-line	27.2	42.6	29.3
VL pre-third-line	4170, log 3.6	78,083, log 4.9	35,802, log 4.55
VL post-third-line	<50 copies/mL	<50 copies/mL	<50 copies/mL

Key: NRTI = nucleoside reverse transcriptase inhibitor; NNRTI = non-nucleoside reverse transcriptase inhibitor; PI = protease inhibitor; II = integrase inhibitor; ABC = abacavir; 3TC = lamivudine; LPV/r = lopinavir/ritonavir; AZT = zidovudine; RAL = raltegravir; ND = not done. * CD4 is expressed as percentage or absolute count.

**Table 2:** Clinical characteristics, third-line regimen and formulations given.

Patient	Age * at Start of Third-Line Regimen	Weight ** at Start of Third-Line Regimen	Regimen/Dose	Actual Formulation Given
RN	44	30	Darunavir 450 mg tablets (3 × 150 mg) BD Ritonavir 80 mg/mL solution 1.25 mL BD Raltegravir 200 mg (2 × 100 mg) chewable tablets BD Zidovudine + Lamivudine 300 mg + 150 mg combination tablet, 1 tablet BD	Crushed Darunavir 3 × 150 mg BD
PGL	24	10.9	Darunavir 200 mg BD Darunavir: Not licensed for use in children < 3 years Ritonavir 32 mg (0.4 mL) BD Lamivudine 6 mL BD Zidovudine 12 mL BD	Crushed Darunavir 3 × 75 mg BD
CB	36	17	Zidovudine 100 mg mane, 200 mg nocte Lamivudine 150 mg OD Darunavir 400 mg tablet BD Ritonavir 100 mg sachet BD Dolutegravir 40 mg BD 4 × 10 mg BD	Crushed Darunavir 1 × 400 mg BD Dolutegravir 1 × 50 mg film coated BD

* months, ** kilograms.

## Data Availability

Not applicable.
